# The importance of belonging for well-being in college students

**DOI:** 10.1371/journal.pmen.0000057

**Published:** 2024-06-13

**Authors:** Isabella Romeo, Harold Stanislaw, Jamie McCreary, Marcus Hawley

**Affiliations:** 1 Kinesiology and Public Health Department and Biological Sciences Department, California Polytechnic State University, San Luis Obispo, California, United States of America; 2 Department of Psychology, California State University, Stanislaus, Turlock, California, United States of America; 3 Materials Engineering Department, California Polytechnic State University, San Luis Obispo, California, United States of America; Xi'an Jiaotong-Liverpool University, CHINA

## Abstract

College students are vulnerable to mental health challenges that include depression, anxiety, and suicidal thoughts. We examined how subjective well-being in 369 college students in the United States was affected by the number friends or family members who could provide essential needs (instrumental support) or with whom intimate or personal matters could be discussed (emotional support), the frequency of engaging with others, satisfaction with these engagements, and the sense of belonging. Engagement satisfaction was affected by emotional support and engagement frequency. Instrumental support had no significant effect, but this could be an artifact of our sample. Emotional support affected belonging, which in turn affected well-being. These results highlight the central role of belonging in establishing and maintaining well-being. Some characteristics may act as well-being risk or protective factors, but these effects are small; all students may experience low levels of well-being. We recommend that institutions monitor the well-being of their students and require participation in curricular and co-curricular activities that are intentionally designed to promote belonging and well-being.

## Introduction

On May 2, 2023, the Surgeon General of the United States stated the country was experiencing an epidemic of loneliness and isolation so severe that the negative health impacts were comparable to smoking 15 cigarettes a day [1]. Connecting with others was described as key to overcoming this epidemic, but establishing and maintaining connections is particularly challenging for college students. The transition from high school requires that students, who are already navigating myriad psychological changes during mid- to late-adolescence [2], adapt to unfamiliar academic and social environments while coping with newfound independence and novel living arrangements [3]. The widespread adoption of virtual instruction since the onset of the COVID-19 pandemic has created new barriers to connecting and increased students’ screen time, which is associated with depression, anxiety, and stress [4, 5]. These factors, coupled with increased sedentary time [6], pressure to succeed, and stress related to academic performance and post-graduation plans [7], contribute to the poor mental health of many undergraduates.

This context sheds light on why one in four incoming freshman college students leave within one year of arriving on campus [8], with a similar proportion diagnosed or treated for depression and anxiety annually [9]. These disturbing trends existed before 2020 but were exacerbated by the COVID-19 pandemic. A meta-analysis found that 31% of college students worldwide reported symptoms of anxiety and 34% reported symptoms of depression during the early stages of the pandemic [10]. Rates varied between countries (partly due to methodological differences), but American college students reported the highest rates of both anxiety (52%) and depression (69%). At one university in the United States, 18% of students had suicidal thoughts [11]. In studies that disaggregated data by gender, males were somewhat more likely than females to report symptoms of anxiety (36% compared to 30%), while females were much more likely than males to report symptoms of depression (56% compared to 34%). More recent research provides strong evidence for higher rates of both anxiety and depression among female college students compared to males [12–14].

A substantial body of research, dating back more than a century to Durkheim’s ground-breaking work on factors contributing to suicide, has shown that mental health challenges are mitigated by social support [15–17]. Among college students, high levels of social support are associated with reduced levels of depression and anxiety [18, 19], lower suicide risk [17], and enhanced life satisfaction and subjective well-being [20, 21].

One form of social support is instrumental support, which is the provision of tangible assistance such as resources and practical help [22]. This enhances the well-being of the provider and the recipient–if the provider is emotionally engaged when supporting the recipient [23]. Students often receive instrumental support from family in the form of financial resources such as tuition and allowances, and from friends who may share food, cars, and even a couch for sleeping. In academic settings, students also receive instrumental support from teachers through the clarification of concepts, correction of misconceptions, elaboration on discussed topics, and demonstrations of problem solving and skill development behaviors. This form of instrumental support can reduce anxiety and increase help-seeking behavior, intrinsic motivation, and effort [22].

Another form of social support is emotional support, which is the provision of care, concern, and empathy [24]. This promotes well-being by providing recipients with a safe space to express feelings and voice fears [25], which helps them cope with stress [26]. Emotional support is also essential for developing a sense of belonging, which involves feeling connections with groups, people, and places. Research shows that belonging is associated with a variety of positive psychosocial outcomes, including improved well-being [27–29].

In college students, increases in belonging are associated with improved psychological well-being [30, 31] and reduced depressive symptoms, even after controlling for other social factors such as loneliness and frequency of social interactions [32]. Belonging does not emerge simply from being around others or having a social support network; engagement with the members of that network through communication or other interactions is also required [33].

Most social support and engagement studies focus on their links to depression and anxiety (e.g. [18]). However, mental health should not be conceptualized merely as the absence of mental illness; subjective well-being (SWB) and other positive experiences are also important (e.g., [34, 35]). SWB is a construct that encompasses life satisfaction, optimism, the absence of negative emotions, and the presence of positive emotions [36]. Increases in SWB are associated with a variety of positive outcomes, including good health, work success, more prosocial behavior, and better relationships with others [37, 38].

Several studies have identified key dimensions that underlie SWB, with belonging emerging as a common theme (e.g., [39, 40]). Research has also demonstrated that SWB is correlated with instrumental and emotional support [23], and with engagement frequency and satisfaction [3]. However, it is not entirely clear if support, frequency, and satisfaction are related to belonging and SWB directly or through intermediate variables. Furthermore, the directionality of these relationships has not been tested; finding a correlation between (for example) belonging and well-being could signify that belonging affects well-being, but it could also signify that well-being affects belonging.

We sought to clarify these issues using a split-sample, structural equation modeling approach [41]. We began by applying exploratory methods to half of our data, which allowed us to develop a structural equation model for predicting SWB from instrumental and emotional support, engagement frequency and satisfaction, and belonging. Structural equation models of this sort have not been previously proposed. Our approach also differed from most other work in this area by considering the number of people providing instrumental and emotional support rather than the level of support.

The model we developed was validated by testing its fit against the second half of our data. Goodness of fit metrics can indicate whether a direct relationship included in the model (i.e., a single-headed arrow pointing from one variable to another) should be dropped because there is no evidence in the data to support it, or whether the model is missing an important mediating variable that should be added. Structural equation models also posit directionality. For example, a model can include an arrow pointing from belonging to well-being (indicating that belonging affects well-being), or in the opposite direction (indicating that well-being affects belonging). Goodness of fit will be better when directionality is veridical.

The validated model that emerged from this approach allowed us to conduct follow-up, exploratory analyses. These aimed to identify variables that could merit further study as potential risk or protective factors for the well-being of students. Several of these variables described living arrangements. Students who live alone might be at greater risk of poor well-being compared to students who live with others, as the solitary home environment might reduce social engagement [42, 43]. If students live with others, their well-being could depend on how they are related to those individuals. Students living with their parents or guardians–as opposed to roommates who may be closer to them in age–may experience lower levels of well-being because they are unable to fully express the independence that many college students desire, and because parents may not recognize and acknowledge the changing demands that college places on undergraduates [44]. Conversely, students who are living with a partner or spouse might experience enhanced well-being, because the relationship (assuming it is functioning well) should provide a positive social environment [45].

We also explored the potential relationship of demographic variables to well-being. The literature provides ample evidence for lower levels of well-being in minoritized students [46, 47]. We lacked information on the ethnicity or race of our participants, but we tested for a main effect of gender (females may have lower levels of well-being than males [48, 49]), a main effect of major (STEM majors may have lower well-being than other students [50, 51]), and a gender by major interaction. The interaction relates to minoritization, because females are underrepresented in STEM and may experience reduced well-being because of this [52, 53].

Finally, we examined the novelty of the student’s social environment in school and at home. In part this involved comparing freshmen to more advanced students, as students transitioning from a familiar high school environment into an unfamiliar college environment might be expected to be at particular risk for well-being [54, 55]. We also compared students who had been in their current living arrangement for 12 months or less to students who had been in the same living arrangement for more than 12 months; the former might be predicted to have reduced well-being while they adapt to a new social environment where they live [56, 57].

## Method

### Participants

The research was conducted under protocols approved by the Institutional Review Boards at California State University, Stanislaus (#S22-02) and California Polytechnic State University, San Luis Obispo (#2021-105-OL). Data were provided by three groups of respondents. Group A (*n* = 101 before screening) consisted of students attending a 4-year public university in California with an open admission policy. Most students attending this institution live off campus–many with their parents. Respondents in this group received extra credit in a psychology course for completing the survey. Group B (*n* = 378 before screening) consisted of students attending a highly selective, 4-year public university in California. Many students live on campus and a substantial majority attend from outside the area. Respondents in this group received no compensation for completing the survey. Group C (*n* = 623 before screening) was recruited from Amazon’s Mechanical Turk (MTurk), which is an online crowdsourcing platform. All Group C respondents indicated they were attending 2- or 4-year institutions in the United States. The two institutions from which Groups A and B were recruited shared similarities that might have reduced the diversity of the corresponding responses. Group C was recruited to survey a wider variety of institutions, rendering the findings more generalizable and increasing the sample size.

All respondents were residents of the United States and 18–29 years old. Older respondents were excluded, as 80% of undergraduates in the United States are younger than 30 [58]. Group C respondents could receive up to US $1.00 for completing the survey, which may have tempted non-students to present as students. Accordingly, criteria were developed to screen out respondents who were considered unlikely to be current students. Respondents were also excluded if they provided evidence of inattentive responding. The S1 File describe these criteria and the number of respondents excluded for each. Table 1 summarizes the characteristics of the retained respondents.

**Table 1:** Characteristics of retained respondents.

Characteristic	Group A (*n* = 73)	Group B (*n* = 220)	Group C (*n* = 76)	All respondents (*n* = 369)
Gender (% female, % male, % non-binary, % not stated)	86.3, 12.3, 1.4, 0.0	62.3, 34.5, 1.4, 1.8	31.6, 65.8, 0.0, 2.6	60.7, 36.6, 1.1, 1.6
% California residents	100.0	83.2	10.5	71.5
Completed semesters (*M* ± *SD*)	4.10 ± 2.53	3.19 ± 2.41	5.29 ± 2.62	3.78 ± 2.60
Current semester units (*M* ± *SD*)	13.88 ± 2.66	10.21 ± 1.59	11.36 ± 4.34	11.16 ± 2.85

The three groups of respondents provided considerable diversity among participants. Groups A and B were predominantly female, while Group C consisted largely of males. Groups A and B consisted primarily of California residents; most Group C respondents lived elsewhere. The three groups also spanned a variety of academic workloads and number of completed semesters. As indicated in the S1 File, Group A contained many social science majors (reflecting their recruitment through psychology classes), engineering was well represented in Group B, and Group C included many business majors.

A broad range of living arrangements was included as well: More than half of Group A respondents lived with their parents; most Group B respondents lived with one or more roommates to whom they were not related; and a large proportion of Group C respondents reported living alone. Respondents also differed markedly in the length of their current living arrangement, with 9.1% having lived in their current arrangement for less than 6 months and 38.2% having lived in their current arrangement for more than 1 year.

### Materials

Data were gathered with an online survey reproduced in the S1 File; only a summary is provided here. The survey was administered by Qualtrics and described as a “Living Situation Survey” on the survey landing page, which presented an informed consent form (see first two pages of survey in S1 File). Respondents consented by clicking on an “I Agree–Begin Survey” button at the bottom of the form. They were then asked to provide their student ID number (Groups A and B) or MTurk worker ID (Group C), their age and gender, and the US state or territory in which they resided along with the corresponding ZIP code.

The next survey page asked how many institutions the respondent was attending. Those who indicated they were not attending any institutions were prevented from further survey completion, while the remainder provided the names of up to three institutions. For each of these, respondents indicated: their major(s); whether the institution used semesters, quarters, or trimesters; the number of academic terms completed at the institution; and the number of units currently being taken. Most Group A respondents and all Group C respondents were also asked to provide the nickname given to students at their institution(s) as a check on respondent self-reports of college attendance. However, this question was a late addition to the survey and was not presented to the first 29 Group A respondents or any Group B respondents.

Next, respondents described their current living arrangement. Among the options provided were living with “my parents or guardians” and living with “a spouse or partner.” For convenience, these are described below as living with parents and living with a partner, respectively.

Social supports were then assessed, using items adapted from the Lubben Social Network Scale [59] and Kaiser Permanente’s Your Current Life Situation Survey [60]. Emotional support was defined as the number of friends or family members with whom the respondent could discuss intimate or personal matters, while instrumental support was defined as the number of friends or family members who would help or provide resources if needed. A 5-point, ordinal response scale (ranging from “none” to “a lot”) was used for both forms of support. Respondents used another 5-point, ordinal response scale (ranging from “never or almost never” to “several times a day”) to describe how often they communicated with close friends who were not family members, with family members, and with others in their household. Engagement satisfaction was assessed by asking “How satisfied are you with the amount of companionship and social interaction you have?” The 5-point, interval response scale ranged from “very unsatisfied” to “very satisfied.”

Next, the survey assessed SWB with two methods presented in random order. One method, the Seity Check-In (SCI), was developed by Seity Health [61]. The SCI asks respondents to rate their energy, direction, belonging, and joy using a 5-point “happy face” response scale. The SCI is a formative rather than reflective measure, so internal consistency is meaningless; however, the SCI has strong criterion and construct validity [62]. The four SCI items were always presented in the same order. The other SWB assessment was the Short Warwick-Edinburgh Mental Well-Being Scale, or SWEMWBS [63]. The seven SWEMWBS items used a 5-point Likert response scale and were presented in random order along with two attention checks: “Today I am 7 years old” and “Today I am completing a survey.” Similar to Davison et al. [64], the SWEMWBS items were reworded to ask how respondents felt “today,” rather than over the previous 2 weeks as in the original instrument. The final survey section presented open-ended questions asking respondents to describe their current mood and the social interactions they had most frequently. (We did not examine the open-ended responses in this study.) A debriefing form followed.

### Procedure

Data were gathered from March 17, 2022 through May 17, 2022. Group A students could access the survey through an online research participation management system and that listed the study under the title “Ever Get Lonely?” Group B students were recruited through emails sent to department chairs and on-campus clubs. The S1 File include a redacted version of this email. Group C participants were recruited through postings on MTurk that described the study as a “brief academic survey [that] asks about your mood and your living situation.” All Group C respondents were required to have previously completed at least 5,000 MTurk postings, with an approval rating of at least 98%. These criteria are commonly applied in MTurk studies to improve response quality (e.g., [65]). Group C respondents received a minimum of US $0.50 for completing the survey. A US $0.25 bonus was awarded for each attention check that was answered correctly, so a maximum of US $1.00 could be earned.

Quarter and trimester units were multiplied by two-thirds to convert them to semester units. As noted in the S1 File, the actual responses were replaced with missing values when respondents reported having completed too many units for their age, or enrolling in too many or too few units.

Responses to several items were combined to reduce the dimensionality of the data. The responses for the frequency of communicating with close friends and with family were averaged to produce an engagement frequency measure. The responses to six of the seven SWEMWBS responses were averaged to yield an abridged SWEMWBS well-being score. “I feel close to other people today” was omitted because it assesses belonging and was examined separately. Similarly, the SCI energy, direction, and joy responses were averaged to provide an abridged SCI well-being score; the SCI belonging response was held out as a separate variable. For some analyses, the SWEMWBS and SCI belonging items were averaged to produce a combined belonging measure, while the two abridged well-being measures were averaged to produce a combined well-being measure.

### Analysis

Responses were downloaded from Qualtrics into Excel version 16.70, where they were cleaned (e.g., to identify duplicate respondents) and filtered (e.g., to determine if the respondent was likely a current student). The respondents retained for analysis were sorted first by group, then by gender, and finally by the Qualtrics response ID (a quasi-random variable). The first half of respondents within each group/gender combination was then assigned to an exploratory sample (*n* = 184; 112 females, 68 males, 2 non-binary, and 2 no gender provided); the remaining respondents were assigned to a confirmatory sample (*n* = 183; 112 females, 67 males, 2 non-binary, and 2 no gender provided). The data were then imported into Stata version 18 for analysis and structural equation modeling (SEM). The S1 File include a complete list of the data processing steps and the Excel and Stata data files (redacted to preserve respondent anonymity).

Items were scored from 1 to 5 (for the lowest and highest scores). Adaptive LASSO [66] was used in the exploratory sample to determine which variables predicted engagement satisfaction, the combined belonging measure, and the combined well-being measure. The predictor variables were emotional support, instrumental support, and engagement frequency. Engagement satisfaction was added as a potential predictor of belonging. Both well-being and belonging were added as potential predictors of well-being. The LASSO methodology assumes that predictors are linearly related to outcomes. Linearity is of potential concern for all variables, but especially for ordinal predictors (such as emotional and instrumental support). Accordingly, for each pair of non-binary predictor and outcome variables we compared the Spearman correlation (which can accommodate nonlinear relationships) to the corresponding Pearson correlation. Linearity is implied when the two correlations are similar.

The variables that emerged as significant predictors (*p* < .05) in the exploratory sample were used to develop an SEM model describing their interrelationships. The SCI and SWEMWBS belonging measures were entered in the SEM model as separate indicators of a latent belonging construct. Similarly, the SCI and SWEMWBS abridged well-being measures were entered in the SEM model as separate indicators of a latent well-being construct. The resulting model was then tested against the confirmatory data, using maximum likelihood for parameter estimation. Model elements (directional arrows signifying that one variable affects another) were retained only if they were statistically significant (*p* < .05) in the confirmatory sample.

After the SEM model had been refined using the confirmatory sample, the parameters were re-estimated from the combined exploratory and confirmatory samples. The resulting final SEM model provides a framework for understanding the relationships between the variables that affect belonging and well-being. It was also used to predict engagement satisfaction, the latent belonging score, and the latent well-being score for each respondent.

Follow-up, exploratory analyses of variance (ANOVAs) were conducted to examine the hypothesized risk and protective factors for well-being. Separate ANOVAs were conducted for each variable retained in the final SEM model, using the levels reported by the respondents rather than those predicted by the SEM model.

A one-way ANOVA with Helmert contrasts was used to compare four mutually exclusive living arrangements: Respondents who lived alone (*n* = 23), those who lived with their parents (*n* = 76), those who lived with a partner but not with their parents (*n* = 35), and those who lived with roommates but not with their parents or a partner (*n* = 233). One respondent lived with their parents and a partner, and another respondent did not indicate their living arrangement; both were excluded from this analysis. The Helmert contrasts were orthogonal and compared respondents who lived alone to those who lived with others, those who lived with their parents to those who lived with others, and those who lived with a partner to those who had roommates but did not live with their parents.

A factorial ANOVA tested for main effects and the interaction between gender and major (STEM or non-STEM). Only respondents who identified as male or female were included in this analysis. Respondents majoring in engineering or a physical science were considered STEM majors, while students in all other majors were considered non-STEM. Respondents who combined a STEM major with a non-STEM major were excluded from this analysis (*n* = 11). Respondents who were undeclared at one institution but had a STEM or non-STEM major at another institution were retained, but were excluded if they were undeclared at all of their institutions (*n* = 7). The resulting design was unbalanced: There were 46 female STEM majors, 62 male STEM majors, 168 female non-STEM majors, and 67 non-STEM male majors.

Another factorial ANOVA tested for main effects and the interaction between class standing (freshman or more advanced) and the novelty or familiarity of the respondent’s current living arrangement. Respondents were considered to have a novel living arrangement if they had resided there for 12 months or less, and a familiar living arrangement if they had resided there for more than 12 months. This design was also unbalanced: There were 103 freshmen in a novel living arrangement, 19 freshmen in a familiar living arrangement, 119 more advanced students in a novel living arrangement, and 117 more advanced students in a familiar living arrangement. Nine respondents were excluded because their class standing and/or the novelty of their current living arrangement was not known.

## Results

### LASSO analyses

All predictors in the LASSO analyses (emotional support, instrumental support, and engagement frequency) appeared to be approximately linearly related to engagement satisfaction, the combined belonging measure, and the combined well-being measure. As shown in Table 2, the Pearson correlations were comparable to the Spearman correlations. Similarly, the Pearson correlations between engagement satisfaction and the combined belonging and combined well-being measures, and between the combined belonging measure with the combined well-being measure, were comparable to the corresponding Spearman correlations. These findings suggest that the predictor variables–despite their ordinal nature–are appropriate for use in LASSO and SEM analyses [67].

**Table 2:** Spearman (ρ) and Pearson (*r*) correlations in the exploratory sample (*n* = 181 to 184).

Predictor	Outcome					
	Engagement satisfaction		Combined belonging		Combined well-being	
	*ρ*	*r*	*ρ*	*r*	*ρ*	*r*
Instrumental support	.22	.20	.16	.23	.12	.20
Emotional support	.35	.36	.31	.34	.32	.35
Engagement frequency	.41	.39	.38	.39	.31	.32
Engagement satisfaction	–	–	.56	.58	.46	.47
Belonging	–	–	–	–	.69	.73

The LASSO results indicated that engagement satisfaction was best predicted by emotional support and engagement frequency (out-of-sample *R*^*2*^ = .19). The combined belonging measure was best predicted by emotional support, engagement frequency, and engagement satisfaction (out-of-sample *R*^*2*^ = .35), whereas the combined well-being measure was best predicted by the combined belonging measure and emotional support (out-of-sample *R*^*2*^ = .53).

### SEM modeling

**Fig 1:**
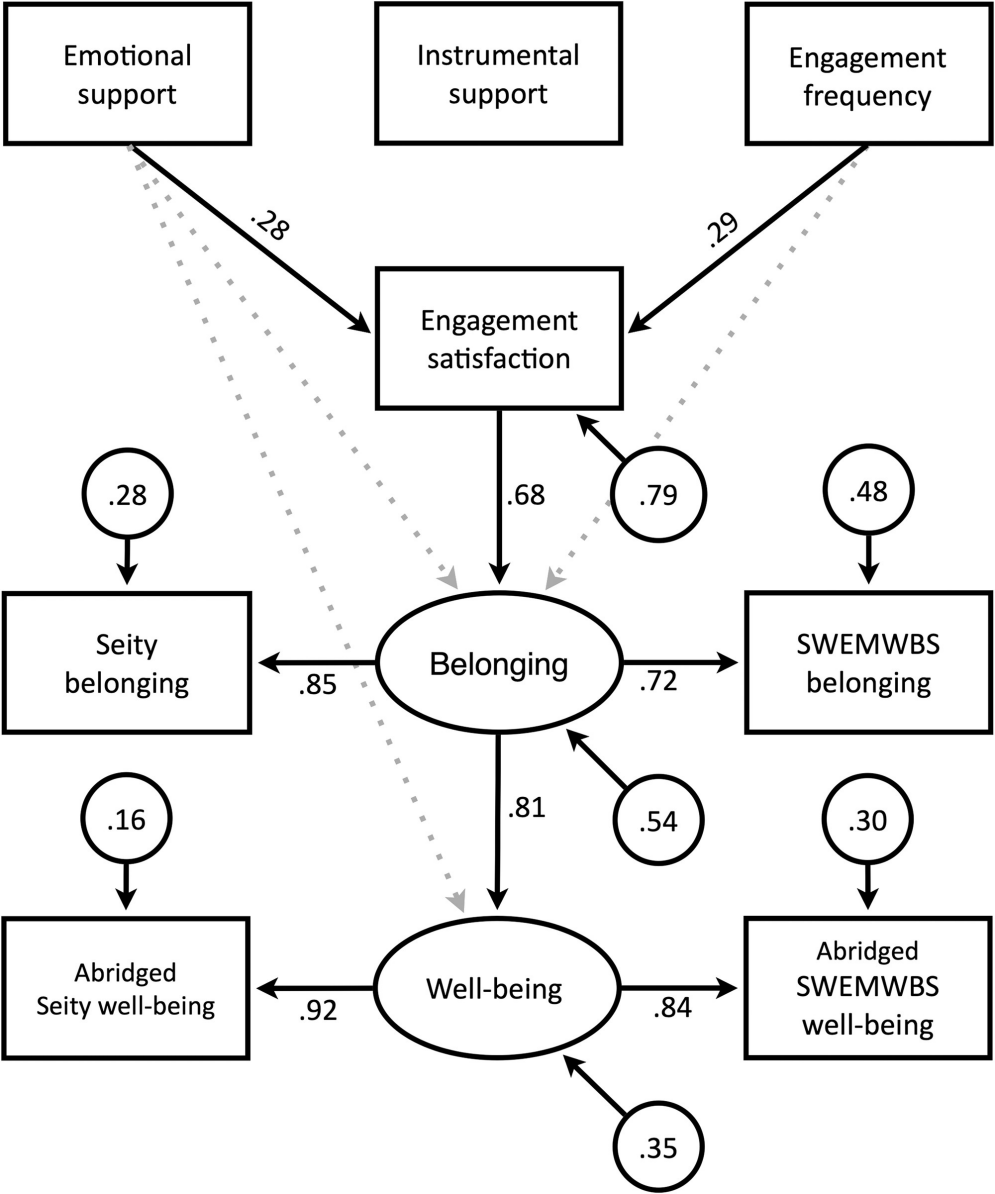
SEM model with standardized coefficient values and error variances. *Note*. Broken lines indicate paths that were significant in the LASSO analysis of the exploratory sample but were not statistically significant (*p* < .05) in the SEM analysis of the confirmatory sample.

The adaptive LASSO findings led to the initial SEM model, illustrated by the solid and broken lines in Fig 1. Following convention, rectangles represent measures that are directly assessed, while ovals represent latent constructs that are not observable but inferred to exist. Variables from which arrows begin can be thought of as independent or predictor variables, while variables at which arrows end can be thought of as dependent variables. The paths indicated by broken lines in Fig 1 were not statistically significant in the confirmatory sample: There was no support for a direct path from emotional support to belonging (*p* = .151) or to well-being (*p* = .880), or from engagement frequency to belonging (*p* = .396). The remaining paths were all significant (*p* < .001).

Deleting the non-significant paths led to the final SEM model, depicted by solid lines in Fig 1. This model cannot be rejected by the confirmatory sample, χ^2^(17) = 18.20, *p* = .376, and three common measures–when compared to criteria recommended by Hu and Bentler [68]–suggest the model fits the sample well. The comparative fit index (CFI) was 0.997 (values ≥ 0.95 are considered good), the standardized root mean squared residual (SRMR) was 0.039 (values ≤ 0.08 are considered good), and the root mean squared error of approximation (RMSEA) was 0.020 (90% CI = [0.000, 0.072]; values ≤ 0.06 are considered good).

The parameter values in Fig 1 were estimated after combining the exploratory and confirmatory samples (*n* = 360 after excluding respondents with missing data). The circles in Fig 1 are error variances, while the numbers along each arrow are path coefficients that describe the strength of the relationship between the two variables connected by the arrow. The parameter values in Fig 1 were derived using standardized scores; raw-score values are available in the S1 File. The chi-square test for the final SEM model is significant when tested against the combined exploratory and confirmatory samples, χ^2^(17) = 51.95, *p* < .001. This suggests the model can be improved, but modification indices (detailed in the S1 File) did not reveal any theoretically meaningful elements that could have been added, and adding elements merely to improve fit should be avoided [69]. Furthermore, the three fit indices suggest that the final SEM model fit the data well in the combined sample: The CFI value was high (0.965), while the SRMR (0.054) and the RMSEA (0.076, 90% CI = [0.053, 0.100]) were both low. These findings imply that the final SEM model is a plausible representation of the relationships between the variables that affect well-being.

The path coefficients in Fig 1 can be interpreted as correlations after partialing out co-predictors. For example, the correlation between emotional support and engagement satisfaction was .28 after partialing out engagement frequency, while the correlation between engagement frequency and engagement satisfaction was .29 after partialing out emotional support. These relatively low values might seem to suggest that emotional support and engagement frequency are weak predictors of engagement satisfaction; however, partial correlations can underestimate the explainable variance and the two variables together predicted engagement satisfaction moderately well (*R*^2^ = .21). The final SEM model predicted belonging (*R*^2^ = .46) more accurately than engagement satisfaction, and well-being (*R*^2^ = .65) more accurately than belonging. The model also generated more accurate predictions for the two SCI measures (*R*^2^ = .72 for belonging and *R*^2^ = .84 for abridged well-being) than the corresponding SWEMWBS measures (*R*^2^ = .52 for belonging and *R*^2^ = .70 for abridged well-being).

### Well-being risk and protective factors

The final SEM model provides insight into the psychological factors that affect well-being. None of the variables in this model are directly observable; they can only be assessed with a survey or similar instrument that provides insights into the respondent’s feelings. An important question is whether other, more readily observed factors are predictive of high or low levels of well-being and the variables that affect these levels.

#### Living arrangement

The ANOVA contrast tests demonstrated that the respondent’s living arrangement has very little relationship to well-being and most of the factors in the SEM model that affect it (Table 3; see S1 File for additional statistics). The largest effect of living arrangement was on emotional support, where it accounted for 8% of the variance. This effect was almost entirely due to the low level of emotional support reported by respondents who lived with their parents compared to respondents who lived with other individuals. Several other statistically significant effects were also observed: Engagement frequency was lower for respondents who lived with a partner; engagement satisfaction was lower for respondents who lived with their parents; and belonging and well-being were higher for respondents who lived with a partner. However, none of these latter effects accounted for more than 2% of the variance in their corresponding measures.

**Table 3:** Means for respondents living alone (*n* = 22–23), living with their parents or guardians (*n* = 75–76), living with a partner or spouse (*n* = 35), and living with other roommates (*n* = 229–233).

Variable	Living alone	Living with parents	Living with partner	Living with other room-mates	*η* ^2^	Living alone contrast *p*	Living with parents contrast *p*	Living with partner contrast *p*
Emotional support	3.09	2.86	3.20	3.48	.08	.645	< .001**	.068
Engagement frequency	3.48	3.63	3.23	3.59	.02	.984	.088	.030*
Engagement satisfaction	3.52	3.58	4.03	3.75	.02	.237	.037*	.132
Mean of combined SCI and SWEMWBS belonging items	3.46	3.66	3.97	3.64	.01	.132	.261	.045*
Mean of abridged SCI and SWEMWBS well-being scores	3.54	3.60	3.86	3.57	.01	.420	.306	.029*

*p < .05

**p < .01

*Note*. The “living alone” contrast compares respondents who live alone with those in the other three groups. “Living with parents” compares respondents who live with their parents or guardians to those who live with a partner, spouse, or other roommates. “Living with partner” compares respondents who live with a partner or spouse to those who live with roommates other than their parents or guardians.

#### Gender and major

The factorial ANOVA of gender and major found no significant interaction for any variable in the SEM model. However, females reported lower levels of well-being than males, and STEM majors reported lower levels of both belonging and well-being than non-STEM majors. Table 4 reports the main effect statistics and the marginal means by gender and major; raw means and interaction statistics are available in the S1 File. As with living arrangement, the effect sizes for gender and major were small, accounting for only 1%-2% of the variance in belonging and well-being.

**Table 4:** Marginal means for females (*n* = 212–214) and males (*n* = 126–129) controlling for major imbalance, and marginal means for STEM majors (*n* = 106–108) and non-STEM majors (*n* = 232–238), controlling for gender imbalance.

Variable	Females	Males	Partial *η*^2^	ANOVA *p*	STEM majors	Non-STEM majors	Partial *η*^2^	ANOVA *p*
Emotional support	3.38	3.28	.00	.356	3.38	3.28	.00	.323
Engagement frequency	3.56	3.49	.00	.499	3.46	3.60	.00	.206
Engagement satisfaction	3.75	3.73	.00	.899	3.64	3.83	.01	.114
Mean of combined SCI and SWEMWBS belonging items	3.62	3.66	.00	.705	3.52	3.76	.01	.034*
Mean of abridged SCI and SWEMWBS well-being scores	3.50	3.71	.02	.019*	3.50	3.71	.02	.017*

*p < .05

#### Class standing and novelty of current living arrangement

The factorial ANOVA of class standing and living arrangement novelty found only one significant effect: Emotional support was lower for respondents in familiar living arrangements than for those in unfamiliar arrangements (see Table 5 and S1 File). This effect was small, as novelty of living arrangement accounted for only 3% of the variance in emotional support.

**Table 5:** Marginal means for freshman (*n* = 120–122) and more advanced students (*n* = 233–236) controlling for stability of living arrangement, and marginal means for students in their current living arrangement for 12 months or less (*n* = 220–222) and more than 12 months (*n* = 133–136) controlling for class standing.

Variable	Freshmen	More advanced students	Partial *η*^2^	ANOVA *p*	In current living arrange-ment ≤ 12 months	In current living arrange-ment > 12 months	Partial *η*^2^	ANOVA *p*
Emotional support	3.25	3.25	.00	.980	3.46	3.05	.03	.001**
Engagement frequency	3.50	3.53	.00	.821	3.57	3.45	.00	.360
Engagement satisfaction	3.58	3.77	.00	.188	3.72	3.63	.00	.532
Mean of combined SCI and SWEMWBS belonging items	3.67	3.70	.00	.800	3.64	3.74	.00	.428
Mean of abridged SCI and SWEMWBS well-being scores	3.47	3.66	.01	.072	3.54	3.59	.00	.653

**p < .01

## Discussion

At academic institutions, belonging is often cited as an important predictor of retention, persistence, and academic success [70]. Our findings point to the centrality of belonging in helping to establish and maintain well-being, which is arguably even more important. Our results align with those reported by Dutcher et al. [32] and Gopalan et al. [71] who found that depression and anxiety are lower in college students who report stronger feelings of belonging.

In our sample, belonging accounted for about two thirds of the variance in well-being. Belonging, in turn, was affected by satisfaction with social engagements, but satisfaction–which is indicative of the quality of social interactions–accounted for less than half of the variance in belonging. This suggests that other factors, separate from those we examined, are important mediators of belonging.

Personality may play a role here. The need to connect with others and belong is universal [27, 28], but individuals differ in the intensity of that need [72]. Another variable that may affect belonging in college is campus climate, which relates to the degree to which students feel respected (or, conversely, ignored or discriminated against) and may be especially important for students from historically underrepresented groups. It should also be noted that, while the sense of belonging is often conceptualized as describing the feelings that students harbor regarding their institution, students may develop multiple and different senses of belonging. For example, they may feel they belong–or don’t belong–to their major, which could include developing a “science identity” if they are STEM majors [73]. Students who are active in Greek life may have a sense of belonging to their fraternity or sorority [74], athletes may have a sense of belonging to their team, residential students may have a sense of belonging to their dorm, and so on.

Our data suggest that instrumental support is unrelated to engagement satisfaction, belonging, and well-being. This contradicts the finding that instrumental support can promote well-being in some conditions [22, 23]. However, the lack of an instrumental support effect may be an artifact of our sample. Almost all respondents who reported their instrumental support had some level of support; only six (1.6%) indicated they had no friends or family members who would help with food, housing, or other essential needs. Furthermore, the institutions attended by respondents in Groups A and B provide students with free access to food pantries, emergency housing, and mini-grants to cover unexpected expenses; the same may be true for many of the Group C institutions. Given these institutional supports, college students may feel little need for instrumental support from friends and family. It could also be that college students feel they have adequate instrumental support if just one or two family members or friends can provide these needs. Most of our respondents (93%) met or exceeded this threshold.

By contrast, our findings strongly suggest that emotional support is an important predictor of engagement satisfaction–and thus an indirect predictor of belonging and well-being. This finding was expected, but we did not anticipate that engagement satisfaction would continue to increase as the number of individuals providing emotional support increases. This latter finding was surprising because as the size of a social support network increases, so do the challenges in maintaining that network by engaging authentically with each member. We did not question our respondents about their use of social media, but it is tempting to interpret our finding as reflecting the pressure that online platform users may feel to continually amass likes and followers [75–77]. Further research in this area could be illuminating.

Engagement satisfaction is fostered by having access to a network of individuals who can provide emotional support, but the frequency of communicating with friends and family also matters. In other words, students must actively participate in their support network to maximize their well-being. It is not clear from our data whether texting, emailing, and other remote forms of asynchronous communication are as effective as phone calls and face-to-face exchanges in promoting engagement satisfaction. It is also not clear whether students regard posting and reading social media as forms of communication that foster engagement satisfaction. Research published to date suggests synchronous engagement is very important (e.g., [78]).

Helping students establish and utilize meaningful support networks can be difficult. It is not sufficient to simply encourage interactions with others, as the resulting engagements may be shallow and do little to foster the development of authentic friendships. Instead, social interactions should be structured intentionally to foster the establishment of deep connections between students. Some instructors attempt to do this in an ice-breaker for online classes that asks students to “share with the class something that is meaningful and explain its significance,” but whether this helps to establish true friendships is not known. What is known is that attraction to others in a university setting can be predicted by both perceived and actual similarity of personal values [79]. Thus, tools that focus conversation on personal values rather than more superficial aspects of the self may prompt the formation of authentic relationships. One potentially useful tool for this purpose is the hierarchical ipsative preference assessment, which allows students to identify their core values in 5–7 minutes [80]. After students complete the assessment, follow-up activities can encourage them to share and explain their values with each other, jump-starting closer relationships.

Sustaining relationships can be challenging, but environments that bring people together in relaxed settings–inside and outside of the classroom–can help. Classes can include intentional opportunities for engagement by going beyond traditional approaches (such as group projects or think-pair-share activities) and scheduling time for students to simply talk with each other and their instructors about non-academic issues. These experiences can improve not just belonging, but also motivation, achievement, and engagement in the course [81]. Additionally, institutions can provide co-curricular activities that are specifically designed to bring students together for social rather than academic purposes. Examples include intramural sports events, as well as game or movie nights. Participation can also be encouraged in student government, clubs, and study groups.

It may be tempting to focus well-being initiatives on students presumed to be at risk of low well-being based on their observable external characteristics. However, we found little evidence to support this practice. In our sample, a student’s living arrangements, gender, major, and class standing had little or no relationship to their well-being. Females and STEM majors reported lower average levels of well-being than males and non-STEM majors, replicating common findings in the literature (e.g., [82]). However, gender and major together account for only about 4% of the variance in well-being. Similarly, our findings suggest that students may experience higher levels of belonging and well-being if they live with a partner than if they do not, but these effects only account for about 1% of the variance in the belonging and well-being. Thus, it is dangerous to concentrate efforts on certain groups of students while ignoring others simply because they belong to a particular demographic group or have a particular background; all students are at risk of low well-being.

## Limitations and future research

Our conclusion regarding risk factors is limited, because we did not ask about ethnicity or race, family educational history, socioeconomic status, or other factors that can generate feelings of isolation in college students. The respondents included 46 female STEM majors, but we do not know how many or which of our respondents were marginalized in other ways. Future research should use targeted efforts to intentionally recruit marginalized students, in order to determine how well the model describes them and whether institutions should take different steps to assure their well-being. For example, freshmen in sexual/gender minority groups may be at especially high risk of experiencing anxiety and depression [83], but only four respondents in our sample identified as non-binary, preventing us from examining the specific concerns and needs of this group.

We also had small sample sizes for some of our analyses. Only 23 respondents reported living alone, 35 reported living with a spouse or partner, and 19 freshmen reported being in their current living arrangement for more than 12 months. Larger and more diverse samples for these groups in particular could be useful. For example, 17 of the 19 freshmen who were in a familiar living arrangement lived with their parents.

This points to another limitation of our study: Many of the potential well-being risk and protective factors are correlated with each other. A balanced study design could help disentangle the effects of each individual factor. However, this would not change our basic conclusion that none of these factors have much influence on well-being or the other variables in the SEM model. Similarly, we did not control for the Type I error rate inflation that resulted from the large number of statistical tests we conducted with our ANOVAs, but doing so would merely render nonsignificant some of the findings we currently describe as significant. Thus, the primary take-away from our study is the SEM model and the relationships it describes between variables that affect well-being, not the degree to which well-being does or does not differ between students with various characteristics.

It is not known how typical our respondents are of American college students in general. We also do not know the motivational factors that led some potential participants to complete the survey and others to skip it. Thus, little emphasis should be placed on the descriptive statistics of the variables we examined, such as the percentage of students in our sample who indicated they have high or low levels of belonging or well-being; a more representative sample is needed for this purpose. However, our sample lacked ceiling and floor effects for the variables of interest and was thus well suited to the correlational analyses required to develop and validate the SEM model.

One implication of the SEM model is that instrumental support is not related to belonging or well-being. We caution against generalizing this finding. Our measure of instrumental support consisted of a single, general question. Furthermore, our respondents may have been biased with respect to institutional support, as the majority attended institutions that provided essential resources. It should also be noted that, during the COVID-19 pandemic–when our data were obtained–individuals who lacked instrumental support may have dropped out of college to cope with other challenges in their lives. This may help explain why so few of our respondents (only 7%) reported that they had no more than one individual who could provide instrumental support.

Our survey included only two belonging items, which were both quite general. Future research could examine whether some senses of belonging are more important than others in determining the well-being of college students, leveraging this information to design effective interventions. It would also be interesting to learn more about the impact of different types of social media usage on emotional support and engagement satisfaction. Much of the research in this area focuses on pre-collegiate students, but a recent article found evidence of an increased risk of depression among college students who spend more than 5 hours per day on their smartphones [84]. This effect could result from reductions in engagement frequency.

Some of the survey items we used were developed exclusively for this study, which is common in this area of research (e.g., [23]). Other items were adapted from existing sources but asked fewer questions about each variable in the SEM model than is typically the case. For example, belonging can be assessed with the 27-item Sense of Belonging Instrument [85], whereas we used only two belonging items. This practice allowed us to limit the effects of survey fatigue while still asking about a large number of factors relating to well-being, as well as detailed questions about each respondent’s academic history and living arrangements. We also modified some items in order to maintain, as much as possible, the same response scale throughout the survey. Future research focused on specific elements within the SEM model could benefit from using published instruments in their original form to provide more nuanced details about those elements. However, despite these limitations, the SEM model we derived accounts for a considerable proportion of the variance in our data and provides solid evidence for goodness of fit.

## Conclusions

One broad implication of our study is that institutions should promote curricular and co-curricular activities that improve belonging. These activities should be a high priority for colleges and universities; well-being is at least as important as traditional learning outcomes. Activities that enhance belonging, such as engagement with student organizations, are crucial to academic success and well-being [86]. Interventions that address student concerns about belonging, fitting in, and struggling in classes have been shown to improve retention and persistence, and are particularly impactful for students in historically underrepresented groups [87].

A second implication is that universities should monitor the well-being of their students. Since our findings suggest that student well-being cannot be readily predicted from external indicators, it must instead be assessed with an instrument specifically designed for this purpose. The SCI tool we utilized in our study may be especially useful here, as it is intended for daily administration. The SWEMWBS is another candidate, although in its original form it should be administered at intervals of 2 weeks or longer.

Finally, we would argue that if institutions truly value the well-being of their students, they should require participation in activities designed to promote it. This would acknowledge that students have limited time, which they allocate to the activities they deem most important. Designating a well-being activity as required, rather than optional, highlights to students the critical importance of self-care.

## Supporting information

S1 File Supplemental materials, including a copy of the survey, anonymized data files, procedures for screening participants, and results from statistical analyses are available at https://osf.io/utcz7/?view_only=20bfd747fba643bfac065b3951483df6. (DOCX)
