# Supplementary material for: The importance of belonging for well-being in college students
Source: PLOS Ment Health. 2024 Jun 13;1(1):e0000057. doi: 10.1371/journal.pmen.0000057 (PMC12798600; doi:10.1371/journal.pmen.0000057)
Supplement: S1 File — (DOCX) [file pmen.0000057.s001.docx]

Supplemental materials, including a copy of the survey, anonymized data files, procedures for screening participants, and results from statistical analyses are available at <https://osf.io/utcz7/?view_only=20bfd747fba643bfac065b3951483df6>.
